# Evaluation of severe malaria case management in Mazowe District, Zimbabwe, 2014

**DOI:** 10.11604/pamj.2017.27.33.11081

**Published:** 2017-05-11

**Authors:** Bargley Makumbe, Cremence Tshuma, Gerald Shambira, More Mungati, Notion Tafara Gombe, Donewell Bangure, Tsitsi Patience Juru, Mufuta Tshimanga

**Affiliations:** 1 Department of Community Medicine, University of Zimbabwe, Harare, Zimbabwe; 2 Ministries of Health and Child Care, Provincial Medical Directorate Mashonaland Central, Bindura, Zimbabwe

**Keywords:** Severe malaria, case management, patient monitoring

## Abstract

**Introduction:**

Malaria is a preventable and curable disease. Mazowe district had been experiencing a lower malaria transmission rate in comparison to other districts in the Mashonaland Central province but it experienced a huge outbreak in the 2013-2014 rainy seasons with a case fatality rate (CFR) of 0.21%. This CFR was the highest in the province and it was twice as much as the national CFR (0.12%) for the same period. We evaluated severe malaria case management in Mazowe district to determine if practice is as per standard treatment guidelines.

**Methods:**

A descriptive cross sectional study was conducted in Mazowe district using the Logical Framework approach. District Health Executives (DHE) members, nurses and severe malaria case notes were purposively and conveniently selected into the study. Key informant Interviews and review of case notes were carried out. All data were analysed using Epi Info 3.5.1.to calculate means and frequencies. Permission to conduct the study was obtained from the Mashonaland Central Provincial Medical Directorate (PMD) Institutional Ethical Review Board (IRB).

**Results:**

The median age in years of the cases was 16 (Q1=7.3; Q3=30.8) and up to 58.1% of the cases were female. Inputs including staff, medicines and medical and laboratory equipment for severe case management were inadequate in the district. Only 60% of severe cases were diagnosed using blood slides and up to 95.6% of cases presented with one or more of the clinical signs of severe malaria. All severe cases were treated using correct anti-malarial and analgesic doses. Patient monitoring was not done as per prerequisite intervals and up to 5% of cases died. The health workers had above average knowledge on severe malaria.

**Conclusion:**

Severe malaria case management inputs were inadequate in the district. For many cases, the district did not follow complicated malaria treatment guidelines for diagnosis, treatment and monitoring. Untrained staff needs training in Severe Malaria Case Management and monitoring of commodity stocks needs to be strengthened.

## Introduction

Malaria is a fatal disease that is both preventable and curable. It is caused by parasites that are transmitted to people through the bites of infected mosquitoes [1]. In the body of a human being, the parasites proliferate in the liver, and then cause infection to red blood cells [2]. The typical symptoms of malaria include fever, headache, and vomiting, and they usually show after 10 to 15 days of a mosquito bite [2]. If treatment is not done, malaria can rapidly become life-threatening by disturbing the supply of blood to vital organs [2]. Plasmodium falciparum is responsible for almost all severe forms of and deaths from malaria [1]. Serious organ failures or abnormalities in the blood or metabolism characterize severe complicated malaria [2]. Patients who present with complicated malaria must be treated with parenteral quinine or intramuscular artemether where available. Severe cases on presentation to a primary health care centre should be given a loading dose of intramuscular or intravenous quinine and then be urgently referred to an admitting institution. In spite of the great efforts towards malaria control in Zimbabwe (which has a largely unstable malaria transmission with 50% of the population living at risk of the disease), malaria is still the third cause of mortality after HIV/AIDS and TB. In 2013, Mashonaland Central province alone contributed 18.7% of the national malaria burden and 11.7% of the national malaria related deaths. Although in comparison to other districts like Mbire, Guruve, Shamva and Mt Darwin, Mazowe district had been experiencing a lower transmission rate in recent years, the district experienced a huge outbreak in the 2013-2014 rainy season and a total of up to 3403 cases was recorded. The district had a case fatality rate (CFR) of 0.21% during the recent malaria season in the first half of 2014. This CFR was the highest in Mashonaland Central province and it was twice as much as the national CFR (0.12%) for the same period. In a Provincial Health Team (PHT) meeting for Mashonaland Central held in mid-September 2014, it was highlighted that Mazowe district did not have adequate quinine for case management during the same malaria season. Moreover, the district fell 1.5% short of their target to do diagnostic tests on all suspected cases during the same period. It was against this background that we set out to evaluate malaria case management (MCM) in Mazowe district. As Mashonaland Central moves towards pre-elimination, adequate case management is a prerequisite complement to epidemic preparedness and vector control (including buffer zone spraying). The findings from the evaluation will help inform the planning and implementation of malaria case management in the district and will also help craft a roadmap towards the target for Zimbabwe to report zero fatality by 2017.

## Methods

A descriptive cross sectional study was conducted using the Logical Framework approach. A review of severe malaria case notes was done in order to assess whether the cases were treated according to the recommended protocol. The study was carried out in Mazowe district at the three referral hospitals namely, Concession District Hospital (CDH), Mvurwi Hospital and Howard Mission Hospital which were selected purposively. Key Informant Interviews were carried out with District health staff (DNO, DMO, Pharmacist) and hospital nurses. These were purposively and conveniently selected. Purposive sampling was used because the knowledge we required in some instances required specific individuals who alone had that kind of knowledge. Such individuals were pharmacy staff and DHE members. Convenient sampling was used in the case of nurses who do case management in the wards. We recruited those who were available in the wards during the period of the study. A total of 94 severe case notes from the period April 2014 to May 2015 were randomly selected into the study from the 3 hospitals. These were captured onto a data abstraction form and then entered into an Epi Info database. Key informant interviews were conducted with the health staff using a questionnaire. All data were analysed using Epi Info 3.5.1. to calculate means and frequencies. Permission to conduct the study was obtained from the Mashonaland Central Provincial Medical Directorate (PMD) Institutional Ethical Review Board (IRB).

## Results

**Demographics**: A total of 40 health workers of median age 35 years and median period on current post 7.5 years were recruited into the study from 2 rural hospitals, 1 district hospital and 1 mission hospital. Most of the participant health workers were female (70 %) and 70 % were nurses. For all the severe cases sampled from the case notes, 15% were aged less than five years and 3.2% were over at least 65 years of age. The median age was however 16 years and up to 58.1% were female. Only 14% of these cases had been referred.

**Case management Inputs**: Across the three hospitals in the district, only environmental health staff were as per staff establishment. Only Howard mission hospital met the recommended staff compliment for doctors while the other three hospitals only met the staff compliment requirements for environmental health. Except at Howard hospital were minimum stock levels could not be calculated, coartem was at least 3.5 times the minimum stock level at the time of data collection. The district hospital did not have any quinine in stock. 5% dextrose was on average 47.0% in short fall for Concession and Mvurwi hospitals. Only the district hospital surpassed the minimum stock requirements for glucometers and glucose test strips Table 1.

**Table 1:** case management Inputs of severe malaria, Mazowe District, Zimbabwe 2014

Input	Type	Concession Hospital Stock Status		Mvurwi Hospital Stock Status		Howard Hospital Stock Status	
		Minimum	Current	Minimum	Current	Minimum	Current
**Medicines**	Coartem	880 blisters	423% above minimum stock	180 blisters	250% above minimum stock	180 blisters	3% above minimum stock
	Oral Quinine	12 tins	out of stock	3 tins	133% above minimum stock	3 tins	100 % above minimum stock
	IV Quinine	600 L	out of stock	300 L	67% below minimum stock	300 L	93% below minimum stock
	Artesunate	200 units	32% above minimum stock	0	Out of stock	0	Out of stock
	Doxycycline	1300 units	150% above minimum stock	900 units	78% below minimum stock	900 units	10% above minimum stock
	Clindamycin	0	out of stock		out of stock		2 tins
	Paracetamol	28 000 units	1954% above minimum stock	6300 units	52% below minimum stock	6300 units	96% below minimum stock
	Diazepam	1 tin	2100% above minimum stock	½ tin	60% below minimum stock	½ tin	800% above minimum stock
	Normal Saline	250 L	out of stock	90 L	out of stock	90 L	Out of stock
	5 % Dextrose	14.5 L	21% below minimum stock	120 L	72% below minimum stock	120 L	92% below minimum stock
**Equipment**	Thermometer	9 units	278% above minimum stock	36 units	67% below minimum stock	36 units	88% below minimum stock
	Glucometer	5 units	20% above minimum stock	2 units	recommended minimum stock	10 units	50% below minimum stock
	Glucose Test strips	1000 units	50% above minimum stock	300 units	92% below minimum stock	1700 units	out of stock
	Microscope	5 units	recommended minimum stock	4 units	recommended minimum stock	2 units	33% below minimum stock
	RDT Kits		recommended minimum stock	250 kits	820% above minimum stock	675 units	-

**Diagnoses of Complicated Cases**: Across all age groups and hospitals, an average of 97.5 % was diagnosed by RDT while an average of 54.6% was diagnosed by microscopy. The greatest proportion of cases tested by Microscopy came from Concession (80.6%), followed by Mvurwi (7.4%) and then Howard (6.0%). By microscopy, 85.9% of the cases tested positive for multi-parasitemia and among the under 5s, only 75.0% were hyper parasitemic. By rapid diagnostic test, 93.5 % tested antigen positive. Up to 95.6% of these cases were diagnosed as complicated malaria, 1.1% was uncomplicated malaria and 3.3% were not specified.

**Treatment and Monitoring of Complicated Cases** : IV Quinine was administered to 78.7% of all the cases of which the loading and maintenance doses were as per guidelines. Oral quinine was administered to 80.9% of all the cases and 94.7% of these had doses as per guidelines. Paracetamol was correctly used among all the 81.9% who got it prescribed as pain killer of choice. Doxycycline and Clindamycin were used as antibiotics of choice among 35.1% and 14.9% of all the cases respectively and in each case, correct doses were administered Table 2. Body temperature, fluid intake and output, BP, blood sugar, pulse and respiratory rate were the most commonly monitored treatment parameters. On average, 46.2% of patients were monitored for these parameters for less than 50% of the recommended time. Temperature, BP and fluid intake were monitored 100% of recommended time among 20.4% only of the patients Table 3. There were 4.4% who were not severe malaria cases but were treated using the severe malaria treatment regimen.

**Table 2:** proportion of severe malaria cases treated according to severe case management guidelines, Mazowe District, Zimbabwe 2014

Medicine	Category	Frequency n (%)
IV Quinine Administered	Yes	74(78.7)
	Can’t Determine	20(21.3)
IV Quinine Dosage	Correct loading dose	74(100.0)
	Correct maintenance dose	74(100.0)
Oral Quinine Administered	Yes	76(80.9)
	Can’t determine	18(19.1)
Oral Quinine Dosage	Correct dose	72(94.7)
Paracetamol Administered	Yes	77(81.9)
	Can’t determine	17(18.1)
Paracetamol Dosage	Correct dose	77(100)
Doxycycline Administered	Yes	33(35.1)
Doxycycline Dosage	Correct dose	33(100.0)
Clindamycin Administered	Yes	14(14.9)
Clindamycin Dosage	Correct dose	14(100.0)
Other Administered	Yes	41(43.6)
Other Administered Dosage	Correct dose	41(100.0)

**Table 3:** proportion of admitted severe cases monitored according to treatment guidelines, Mazowe District, Zimbabwe 2014

Monitoring parameter	Category	Frequency n (%)
Temperature	All intervals	6(12.2)
	Random intervals	43(87.8)
Fluid Intake	All intervals	7(38.9)
	Random intervals	6(33.3)
RBS	All intervals	2(10.0)
	Random intervals	18(90.0)
BP	Random intervals	17(100.0)
Pulse	Random intervals	12(100.0)
Respiratory Rate	Random intervals	13(100.0)

**Treatment Outcomes**: At the end of admission the well and discharged were mostly among those aged 64 years and above and those aged 5 to 64 years (3 of 3) and those aged 5 to 64 years (91.0%). However, 4 of the 5 who died were aged 5 to 64 years. About 94% of severe malaria cases who were admitted within one day of falling sick got well and discharged and none of them died. 88% of severe malaria cases who were admitted after one day of complaining got well and discharged but 8.9% of them died Table 4. Of the 5 who died, all presented with GBW or prostration, 3 with fever and 2 were unconscious. None of them presented with convulsions, pallor or produced dark urine. Among those who died, temperature was the most monitored parameter, never the less only one of the 4 monitored was monitored 100% of the recommended time. BP was monitored < 50% of the recommended time among 3 of the 5. RBS was monitored among 3 of the 5 but monitored > 50% of the time in only 1 of the 3. Respiratory rate was monitored only in 1 of the 5 Table 5.

**Table 4:** proportion of severe cases with positive treatment outcome, Mazowe District, Zimbabwe, 2014

Variable	Category	Outcome	Frequency n (%)
Age	< 5	Well discharged	10(83.3)
		Unwell discharged	1(8.3)
		Dead	1(8.3)
	5 – 64	Well discharged	61(91.0)
		Unwell discharged	2(3.0)
		Dead	4(6.0)
	64 +	Well discharged	3(100.0)
		Unwell discharged	0(0.0)
		Dead	0(0.0)

**Table 5:** patient monitoring procedures for severe malaria cases that died, Mazowe District, Zimbabwe, 2014

Monitoring Parameter	Category	Frequency n/N
Temperature	All intervals	1/5
	Random intervals	3/5
Fluid Intake	All intervals	1/5
	Random intervals	2/5
BP	All intervals	0/5
	Random intervals	3/5
RBS	All intervals	0/5
	Random intervals	3/5
Pulse	All intervals	0/5
	Random intervals	2/5
Respiratory Rate	All intervals	1/5
	Random intervals	0/5

**Health Worker Knowledge on Signs and Complications of Severe Malaria**: Fever, pallor and rigors were most mentioned as signs of severe malaria while on the other end convulsions and prostration were the least stated signs by the participants. All the participants recognized anaemia as a complication of severe malaria while renal failure was least recognized as a severe malaria complication among 75% of participants. Pregnant women and children under 5 years of age were most recognized as high risk groups among the health workers. At 96.9 % of respondents IV Quinine was most recognized as medicine for treating severe malaria while ASAQ, at 25% of participants was least stated as medicine for treating severe malaria Table 6.

**Table 6:** health worker knowledge on high risk groups and medicines for severe malaria, Mazowe District, Zimbabwe, 2014

Variable	Category	Frequency n (%)
High Risk groups	Pregnant women	31(96.9)
	Children < 5yrs	28(87.5)
	HIV positive individuals	25(78.1)
	Travelers from non-malarias areas	21(65.6)
	Treatment failures	19(59.4)
	Sickle cell	17(53.1)
	Elderly	1(3.1)
	Malnourished children	1(3.1)
Treatment of severe malaria	IV Quinine	31(96.9)
	Doxycycline	18(56.3)
	Clindamycin	14(43.8)
	Oral Quinine	11(34.4)
	Artesunate Amodiaquine	8(25.0)

## Discussion

The anti-malarial drug Quinine was relatively below minimum stock status throughout the district except at Mvurwi hospital where it was 133% above minimum stock requirements. Correct loading and maintenance doses of IV Quinine adjusted to age and body weight were administered to all patients to whom it was prescribed. Admitted cases were mostly monitored for Temperature, BP, and Fluid intake and output. Patient monitoring for all necessary parameters was however not done as recommended for patients in admission. About 90.4% of the patients got well and were discharged. Those who died presented at least one day after commencement of complaints. The health workers were able to state correctly most of the signs and complications of severe malaria as well as some of its high risk groups and its treatment. At the point of the study, the stock status for severe malaria medicines was not uniformly distributed among the hospitals in the district. A key thing to note is that the pharmacy at Concession Hospital (the district hospital) also stocks for the district. When medicines have run out at the district hospital, it means either that they have been distributed to the respective health centres in the district or they have literally just run out of stock. The district hospital alone had artesunate and Artesunate-Amodiquine (ASAQ) in stock as it hadn't been distributed to the other hospitals in the district.This was because the staff at those hospitals had not yet been trained in administration of these medicines. The dispensary assistant at Howard Mission Hospital had not been oriented in calculation of minimum stock requirements so it was difficult to measure the availability of medicines at this mission hospital. Since most malaria cases were non-complicated and the district had been experiencing an outbreak, the stock status for coartem was well above minimum stock requirements by at least 350% across the 3 hospitals, Concession, Mvurwi and Rossa. Zurovac D et.al, 2014, found that avoidance of stock-outs causes major improvement to implementation of case management at hospital level [3]. Similarly, in building a strong stock status as a key readiness factor, severe malaria case management in Mazowe will improve.

Most tests were done at the hospitals using RDTs (97.5%) but only 54.6% by Microscopy. Concession hospital was able to do blood slides for all its cases and was also able to do serial MPS tests for 81.8% of all those who had severe malaria and were admitted. Concession hospital being both a referral and district hospital tends to secure more of the laboratory reagents for its broader clientele before distributing them to the other hospitals. Mvurwi and Howard hospitals only carried out tests for 83.8% and 44.4% respectively of all severe malaria cases evaluated. Rossa hospital does not admit patients except prior to referrals out. Only those with a 2+ and above MPS result are technically considered hyperparasitemic. All those who have hyperparasitemia are considered to be severe cases according to the case management guidelines and these were 60.0% of all clients. The rest (40.0%) of the severe cases were not hyperparasitemic and were diagnosed symptomatically and by RDT confirmation. RDTs seem to be a reliable diagnostic method even under emergencies. Similarly, in Malawi Steinhardt L et.al, 2014, the improvement of diagnosis was found to be linked to the cost effective RDTs [4]. Moreover, other studies have found rapid diagnostic tests (RDTs) to be one of the diagnostics that have the largest impact on malaria control today [5]. All treatment constituted of an antimalarial, an antibiotic and an analgesic. The severe malaria treatment guidelines recommend Artesunate for an IV drug and where it's not available, Quinine. Treatment of severe cases was done mainly using Quinine as the antimalarial of choice (78.7% of cases). Of those who were on IV Quinine, 80.9% switched to Oral Quinine which is more preferable for patients who can ingest and not vomit. Artesunate and ASAQ were not used because the health workers hadn't been trained on how to administer them. These are recommended for use under the new case management guidelines except when not in stock which was the case at Howard, Mvurwi and Rossa hospitals. Paracetamol was the main painkiller of choice, prescribed to 81.9% of cases. Clindamycin (14.9%) and Doxycycline (35.1%) were the major antibiotics prescribed but the former was not in stock at any of the hospitals. All the clients had to purchase it at local pharmacies, which is a common trend in resource limited settings like in Uganda, where according to Achan J et al (2011), 44% purchased own medicines and 76% purchased own medical supplies [6]. All doses for all medicines were as recommended per treatment guidelines. Over treatments with antimalarials have been found in instances were diagnosis is either not done or where diagnosis has given false positives. In this case, 3.3% were wrongly treated which is quite like the observation by Sserwanga A et al (2011), in sentinel surveillance where wrong treatments at the end of a malaria surveillance interval were 5% [7].

Regular 4 hourly intervals are meant to be used to monitor different Temperature, Pulse, Blood Pressure, Respiratory rate and Blood sugar. The nursing staff at the clinics either did not do meticulous recording on the patient monitoring charts or were not monitoring patients 100% of the time they should have. The parameter most monitored was temperature with 69.4% of patients monitored 50%-99% of the recommended intervals. A patient is meant to remain in care until their condition stabilizes. The purpose of monitoring a patient during admission is to ensure tracking of the recuperation process and to be able to promptly respond to abnormalities as they emerge in the progression of sickness. Inconsistent monitoring may lead to inappropriate patient care that can lead to the worsening of patient condition or even fatality. Of the 5 patients who died, none of them had had a 100% monitoring. Poor adherence to patient treatment and management guidelines was noted to be associated with poor outcomes by Chanda-Kapata P et al (2011) [8]. The fatality among the investigated severe malaria cases was 5.6%. The transfer rate was 3.4% and the treatment success rate was therefore 91.0%. The 91% recovered and got discharged. Those who died all came and presented with complaints that had lasted for at least 2 days before admission and they all presented with prostration or general body weaknesses. Three of the five who died additionally presented with fever. Temperature and random blood sugar could have been monitored at every interval for these cases to facilitate treatment. Four were in the age group 5 to 64 years. Three of the five had travelled from non-malarias places. Treatment was most successful among those who presented early at the hospital which according to Bartoloni A et al (2012) allows for prompt diagnosis and appropriate treatment that are both crucial in prevention of morbidity and fatal outcomes [9].

**Study Limitations**: We used convenience sampling and we could not reach the calculated sample size as our study participants were attending to patients in the wards. This might have caused under representation of the target population.

## Conclusion

Inputs including Staff, Medicines and Medical Equipment for Complicated Case Management were inadequate in the district. Only 60% of severe cases were diagnosed using the gold standard blood slides and only 95.6% of cases presented with one or more of the clinical signs of severe malaria. All severe cases were treated using correct anti-malarial and analgesic doses. Patient monitoring was not done per prerequisite intervals. Up to 91% of cases recovered and got discharged and 5 % died. Most health workers knew at least one high risk group for severe malaria, clinical signs of and treatment of severe malaria. We therefore recommended training and mentorship of clinicians in severe malaria case management according to new guidelines and strengthening of pharmacovigilance for rigorous commodity stock level management.

### What is known about this topic

- Mortality from untreated severe malaria (particularly cerebral malaria) approaches 100%. With prompt, effective antimalarial treatment and supportive care, the rate falls to 10-20% overall. Within the broad definition of severe malaria some syndromes are associated with lower mortality rates (e.g. severe anaemia) and others with higher mortality rates (e.g. acidosis). The risk for death increases in the presence of multiple complications.

### What this study adds

- In addition to inputs (Staff, Medicines and Medical Equipment) for Complicated Case Management, patient monitoring is very key to good prognosis.

## Competing interests

The authors declare no competing interests.
